# Iodine Deficiency in a Study Population of Norwegian Pregnant Women—Results from the Little in Norway Study (LiN)

**DOI:** 10.3390/nu10040513

**Published:** 2018-04-20

**Authors:** Lisbeth Dahl, Maria Wik Markhus, Perla Vanessa Roldan Sanchez, Vibeke Moe, Lars Smith, Helle Margrete Meltzer, Marian Kjellevold

**Affiliations:** 1 Institute of Marine Research (IMR), P.O. Box 1870 Nordnes, 5817 Bergen, Norway; maria.wik.markhus@hi.no (M.W.M.); jovanelssa@hotmail.com (P.V.R.S.); marian.kjellevold@hi.no (M.K.); 2 Department of Clinical Medicine, University of Bergen, 5020 Bergen, Norway; 3 Department of Psychology, University of Oslo, 0317 Oslo, Norway; vibeke.moe@psykologi.uio.no (V.M.); lrssmth@gmail.com (L.S.); 4 Division of Infection Control and Environmental Health, Norwegian Institute of Public Health, 0456 Oslo, Norway; hellemargrete.meltzer@fhi.no

**Keywords:** urinary iodine concentration, iodine to creatinine ratio, supplement, milk and dairy products, seafood, iodine status, pregnant

## Abstract

Iodine sufficiency is particularly important in pregnancy, where median urinary iodine concentration (UIC) in the range of 150–250 µg/L indicates adequate iodine status. The aims of this study were to determine UIC and assess if dietary and maternal characteristics influence the iodine status in pregnant Norwegian women. The study comprises a cross-sectional population-based prospective cohort of pregnant women (Little in Norway (LiN)). Median UIC in 954 urine samples was 85 µg/L and 78.4% of the samples (*n* = 748) were ≤150 µg/L. 23.2% (*n* = 221) of the samples were ≤50 µg/L and 5.2% (*n* = 50) were above the requirements of iodine intake (>250 µg/L). Frequent iodine-supplement users (*n* = 144) had significantly higher UIC (120 µg/L) than non-frequent users (75 µg/L). Frequent milk and dairy product consumers (4–9 portions/day) had significantly higher UIC (99 µg/L) than women consuming 0–1 portion/day (57 µg/L) or 2–3 portions/day (83 µg/L). Women living in mid-Norway (*n* = 255) had lowest UIC (72 µg/L). In conclusion, this study shows that the diet of the pregnant women did not necessarily secure a sufficient iodine intake. There is an urgent need for public health strategies to secure adequate iodine nutrition among pregnant women in Norway.

## 1. Introduction

A population’s dietary habits and its access to sources of iodine—such as seafood, milk and dairy products, iodized salt, and iodine containing supplements—are important for securing sufficient iodine intake. The frequency and quantity of consumption of these iodine sources are critical determinants of the individual’s iodine status. Iodine is essential for thyroid hormone production, which plays a crucial role in ensuring optimal growth and brain development of the fetus and child. Insufficient iodine intake in utero and during early childhood may damage the developing brain, leading to the loss of intellectual potential, making it one of the most important preventable causes of brain damage worldwide [1]. Suboptimal iodine nutrition gives rise to concern since emerging evidence suggests that children of women with an urinary iodine to creatinine ratio (UI/Cr) <150 µg/g in pregnancy are more likely to have lower scores on verbal IQ, reading accuracy, and reading comprehension at eight years of age [2]. Results from the follow-up of the gestational iodine cohort (9-year and 15-year) in Australia indicate that even mild iodine deficiency during pregnancy may have consequences for the child that are not improved by sufficient iodine intake during childhood [3,4]. Adequate maternal iodine status is therefore especially important since the fetus is dependent on maternal transfer of thyroid hormones in early gestation and supply of iodine later in gestation. The recommended intake of iodine during pregnancy is 175 µg/day in the Nordic countries [5] and 250 µg/day according to World Health Organization (WHO) [6]. 

The urinary iodine concentration (UIC) captures the total iodine intake and is regarded as a reliable biomarker for assessing recent iodine intake at a group level [7]. A median UIC in the range of 150–250 µg/L indicates adequate iodine nutrition in a population of pregnant women [8]. By using a median cutoff of 150 µg/L, insufficient iodine status has been demonstrated in pregnant women in several European countries during recent years [2,9,10,11,12]. The population in Norway has long been considered iodine sufficient due to the contribution of iodine from milk and dairy products. The Norwegian Mother and Child Cohort Study (MoBa), including more than 80 thousand pregnant women, has challenged this assumption as a large proportion of these women was at risk of suboptimal dietary iodine intake [13]. Studies assessing UIC from pregnant women in Norway confirm the findings of insufficient dietary iodine intake [13,14,15]. Further, the MoBa study has shown that an estimated dietary iodine intake <160 µg/day during pregnancy was associated with symptoms of child language delay, behavior problems, and reduced fine motor skills at age three years [16]. 

Both season of the year and time point of urine sampling have been shown to influence the UIC [17,18]. Studies also suggest that gestational week is of importance for the interpretation of UIC in pregnancy [19,20]. With urine samples and dietary data from approximately one thousand pregnant women, we aim to investigate their iodine status by measuring UIC and assess if maternal characteristics and dietary factors may influence the iodine status. Further, we ask if gestational week, time of urine sampling, season of the year, and creatinine levels may influence the interpretation of iodine nutrition in pregnancy.

## 2. Materials and Methods 

### 2.1. Study Population and Design

**Figure 1:**
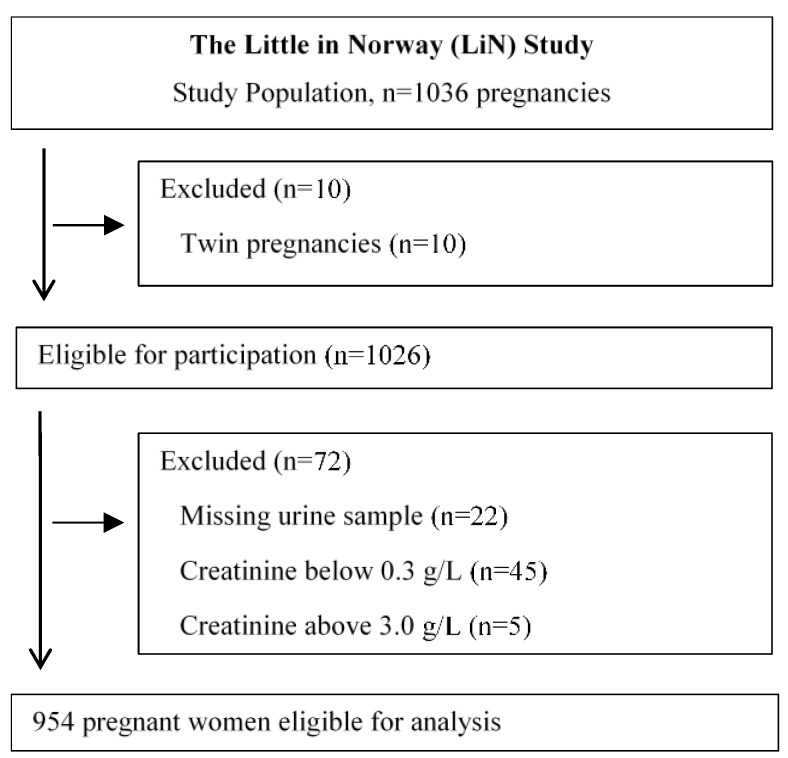
Flowchart of study participants in the current study.

This study used samples and data collected as part of a population-based prospective cohort designed to investigate pre- and postnatal risk factors influencing developmental malleability from pregnancy to 18 months of age. Families were recruited from nine different public health clinics across Norway. The clinics were chosen after considering demographic characteristics and size of the population to include participants from both cities and rural districts with a wide distribution of socioeconomic conditions. At each site, one midwife was trained as a research assistant and invited all the pregnant women at 16–26 weeks gestation to participate, but some women were enrolled before week 16 and some as late as week 30. Between September 2011 and mid-October 2012, 1041 families were enrolled. Five families withdrew their consent, leaving us with 1036 mothers. Three families were excluded due to stillbirth, and 26 were excluded for other reasons before birth of the child (i.e., poor parental health, family moving out of the area, participation felt to be too time consuming). These families let us keep the data we had already collected. We were left with 1007 mothers who gave birth to 1017 children (including 10 twin pairs; multiple pregnancies other than twins have been excluded). Figure 1 shows participation flowchart. 

Data collection phases comprised five points in pregnancy, birth, and four follow-up points up to age 18 months. Data collection up to 18 months was finished by November 2014. In this article urine samples, dietary data, and demographic data from the pregnant women are used. The study was conducted according to the guidelines in the Declaration of Helsinki and was approved by Regional Committees for Medical and Health Research Ethics in Norway (REK 2011/560). Written informed consent was obtained from all subjects.

### 2.2. Data Collection and Categorization of Variables

A non-fasting urine sample was obtained from every pregnant woman in specimens collected at each site. Gestational week, time of the day, and date of urine sample were registered. Since women were enrolled in the study at different gestational weeks, the spot urine sample was categorized into first (0–13 weeks of gestation), second (14–27 weeks of gestation), and third (28–40 weeks of gestation) trimester. The time of day for sampling of the urine was categorized into morning (6–9 a.m.), noon (10–13 a.m.), afternoon (14–17 p.m.), evening (18–24 p.m.), and night (0–5 p.m.). The date of sampling was divided into four seasons; spring (March–May), summer (June–August), autumn (September–November), and winter (December–February). 

A comprehensive questionnaire package was given to mothers and fathers at the first meeting after inclusion, which could take place from pregnancy week 16 to 34, depending on the specific time of enrollment. All parents were asked to answer these questions, irrespective of exact time of inclusion. Among the many questions, data on maternal (and family) characteristics such as age, weight before pregnancy, height, parity, education, socio-economic status, and use of tobacco were obtained. Age of participating women was divided in four categories; <25 years, 25–29 years, 30–34 years, and ≥35 years. The residence of the women was divided into north, mid, west, and east regions of Norway. The north area included women from one sampling site (Tromsø), mid from three samplings sites (Trondheim), west (Bergen) from two sampling sites, and the east area of Norway included three different sampling sites (Oslo). Parental education was categorized into primary and high school (low), University College or University ≤4 years (middle), or University College or University ≥4 years (high). The income of the mother was categorized as <300,000 NOK, 300,000–449,000 NOK, and ≥450,000 NOK. 

Dietary data were collected using a web-based semi–quantitative food frequency questionnaire (FFQ) covering the last three months intake of iodine rich foods, such as seafood, milk, dairy products, and eggs in addition to some questions about the intake of other main food groups, such as fruit and vegetables, bread and cereals, and meat and meat products [21,22]. The first question regarding milk and dairy products asked if they consumed milk and dairy products daily or weekly (‘yes’ or ‘no’). A second question concerned portions of milk and dairy products intake per day, which was categorized as 0–1 portions/day, 2–3 portions/day, and 4–9 portions/day. As to the first question, the non-daily users were included in the category 0–1 portions/day. The seafood intake was recoded to estimate the weekly intake for dinner and the daily intake of seafood as bread spread. Follow-up questions regarding type of seafood were asked to differentiate between lean fish, fatty fish, and the frequency of use of processed fish products (i.e., fish cakes, fish fingers, etc.). The weekly intake of seafood for dinner was then dichotomized into <2 portions/week and ≥2 portions/week. Several questions regarding the use of supplements were asked, answers were divided into non-iodine containing supplement and iodine containing supplement based on information given in several questions. Supplement use was then dichotomized as users (≥5 times/week) and non-users (<5 times/week). The level of iodine in supplements on the Norwegian marked varies from 50 to 225 µg. The maximum level to be added is set to 225 µg iodine per daily dose.

### 2.3. Laboratory Analysis

The spot urine samples were stored at minus 20 °C approximately one month at each sampling site before all samples were shipped to Institute of Marine Research (IMR) and stored at minus 20 °C until analysis of iodine and creatinine concentrations was carried out. Prior to analysis, the urine samples were defrosted in a refrigerator. For the determination of iodine, 500 µL urine was diluted in 4.5 mL 1% tetrametylammonium hydroxide (TMAH) and filtered using a sterile membrane filter with a 0.45 µm pore size and a single use syringe. Samples were analyzed against a urine calibration curve (standard addition curve) to measure the unknown iodine concentration (^127^I) in the collected urine samples. The accuracy of the results was verified with certified reference material; Seronorm Trace Elements Urine (Nycomed Pharma, Asker, Norway) with certified iodine content of 84 µg/L (range 72–96 µg/L) and 304 µg/L (range 260–348 µg/L). UIC was determined by inductively coupled plasma mass-spectrometry (ICP-MS). 

For the urinary creatinine determination, the urine samples were defrosted at room temperature and centrifuged in an Eppendorf (5810R) centrifuge (15 min, 2000× *g* and 4 °C). An aliquot of 200 µL was transferred to the test tube and placed in the Maxmat carousel. All sample treatment was automatic; samples were diluted 1:20 with distilled water and reagents added. Determination of creatinine was performed by a colorimetric enzymatic principle using a MAXMAT PL II multidisciplinary diagnostic platform using the Creatinine PAP kit, (ERBA Diagnostics, Montpellier, France). The method was calibrated with one standard and further controlled with two independent controls.

Creatinine concentration was also used to determine whether the spot urinary sample was valid. We have excluded urine samples with creatinine concentration outside the WHO guidelines of <0.3 g/L and >3.0 g/L [23].

### 2.4. Statistics

We report iodine status in two ways; as the urinary iodine concentration (µg/L) and as the urinary iodine per gram creatinine (µg/g). Both measures were used to explore relations with participants’ characteristics and dietary intake by using independent samples Mann–Whitney U Test. Medians and 25th and 75th percentiles are reported for all these variables (Table 1). The 24-h excretion of iodine (UIE) was estimated by multiplying the iodine-to creatinine ratio by the expected daily excretion of creatinine 1.23 [24]. Spearman’s correlations were calculated for UIC, maternal characteristics and intake of iodine rich foods. Variables that showed a relation with the UIC and iodine to creatinine ratio in the unadjusted analysis (Table 1) were entered in the linear regression model to evaluate associations between maternal characteristics or dietary factors (Table 2 and Table 3). The relationship between UIC or IU/Cr ratio was explored by linear regression, and we used a standard multiple regression model. Two-tailed *p*-values < 0.05 were considered statistically significant. The statistical software package Statistical Package for the Social Sciences (SPSS^®^ Statistics Version 24) was used for all data analyses.

## 3. Results

A total of 954 pregnant women were included in this study, which corresponds to 92.1% of the women consenting to participate (Figure 1). Median age at the time of enrollment was 30 years, with a range of 17–43 years, median weight before pregnancy was 64 kg, with a range of 42–158 kg, and the median height was 167 cm, with a range of 130–184 cm. Median gestational week for the collection of urine sample was 25 (second trimester), with a range of 9–34 weeks.

### 3.1. Urinary Iodine Distribution

**Figure 2:**
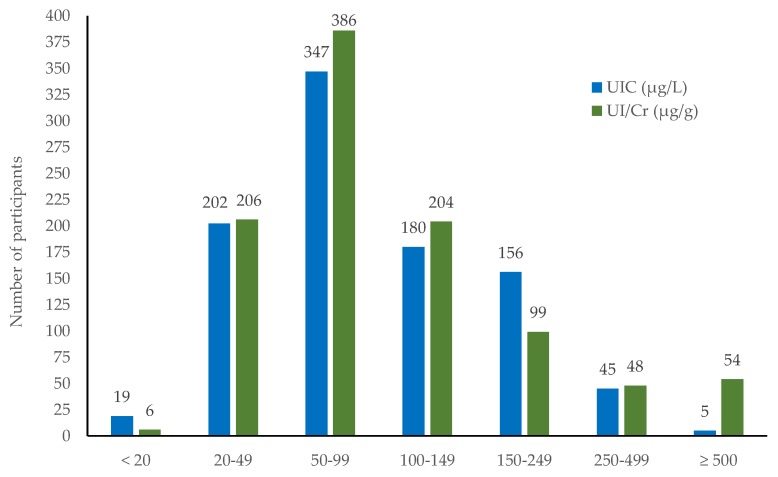
Distribution of spot urinary iodine concentration (UIC) (µg/L) (blue bars) and urinary iodine to creatinine ratio (UI/Cr) (µg/g) (green bars) in the study population (*n* = 954) according to WHO epidemiological criteria [8].

The median UIC and the UI/Cr was 85 µg/L and 82 µg/g, respectively. The overall UIC varied from 5 to 770 µg/L and 78.4% of the pregnant women (*n* = 748) had an UIC less than 150 µg/L. The overall UI/Cr varied from 11 to 1196 µg/g and 84.2% (*n* = 802) had IU/Cr below 150 µg/g. The estimated daily urinary iodine excretion (UIE) was 101 µg/day, with a range of 14 to 1471 µg/day. The distribution of UIC and UI/Cr in the total study population is shown in Figure 2. 

### 3.2. Urinary Iodine Distribution in Relation to Maternal Characteristics

Table 1, Table 2 and Table 3 presents the UIC (µg/L) and UI/Cr (µg iodine/g creatinine) with 25th and 75th percentiles according to maternal demographic, behavioral, and dietary intake characteristics. Maternal residence, season of urine sampling, intake of dairy products and use of supplements differed significantly in the study group (Table 2 and Table 3). These variables were all significantly associated with both UIC and UI/Cr in a non-parametric test. 

Pregnant women living in mid-Norway had significantly lower UIC than women living in north, west or east-Norway. The UI/Cr was significantly higher in women living in east-Norway compared with the three other areas (north, mid, and west). Samples collected during autumn (September–November) yielded significantly lower UIC compared with winter (December–February) and spring (March–May) samples. The UI/Cr values showed a different pattern, as the winter samples were significantly higher than summer and autumn samples. Trimester, time (hour) at urine sampling, smoking, parity, education, and income were all significantly associated with UI/Cr (*p* < 0.01) but not with the UIC. Most of the samples were collected in the second trimester; however, IU/Cr was significantly higher in third trimester compared to first and second trimester. UI/Cr was significantly lower in morning and noon samples compared to afternoon and evening/night samples. Furthermore, UI/Cr was significantly lower in mothers with two or more children compared with non-parous and mothers with one child. Mothers with highest education (≥4 years college/university education) and mothers with highest income (≥450,000 NOK) had higher UI/Cr compared to mothers with lower education and income, respectively. 

The intake of milk and dairy products was more important for the iodine status than the seafood intake as 95.4% of the mothers consumed dairy product daily. The majority (65.2%) of the women reported eating 2–3 dairy portions per day, 14.3% reported an intake of 1 portion per day, and 14.9% reported an intake of 4–6 portions of milk and dairy products per day. Only 1.1% ate 7–9 portions of dairy products per day. The questionnaire did not distinguish between milk, cheese and yoghurt intake, only between portions of intake per day. Of the 4.6% (*n* = 38) non-daily users of milk and dairy products, we were not able to distinguish between non-users and weekly users of milk and dairy products. However, when asked about foods they avoided, eight of the participants reported no consumption of milk and dairy products. Both the UIC and UI/Cr were significantly higher in women consuming ≥2 portions of dairy products as compared with women consuming 0–1 portion/day (Table 1).

The frequency of seafood intake for dinner (*n* = 823) was 1–3 times per month or less among 27.8%, once per week among 42.1%, 2–3 times per week for 28.3% and ≥4 times per week among 1.6% of the women during pregnancy. Consumption of seafood as spread (*n* = 823) showed that 59.2% consumed it seldom, 30.4% 1–2 times/week, and 10.4% consumed seafood as spread ≥3/week. As regards egg intake, 46.9% reported consuming ≤1 egg per week, 11.9% reported consuming 2–3 eggs per week, and 2.7% ate ≥4 eggs per week. 

Use of any type of supplement was reported by 48% at enrollment, however only 15.1% of the women were categorized as users of iodine supplement (i.e., ≥5 times/week). The most frequent used supplement was omega-3 capsules. Both UIC and UI/Cr were significantly higher among iodine-containing supplement users compared to non-users (Table 3).

### 3.3. Assignments between UIC and UI/Cr Ratio

**Figure 3:**
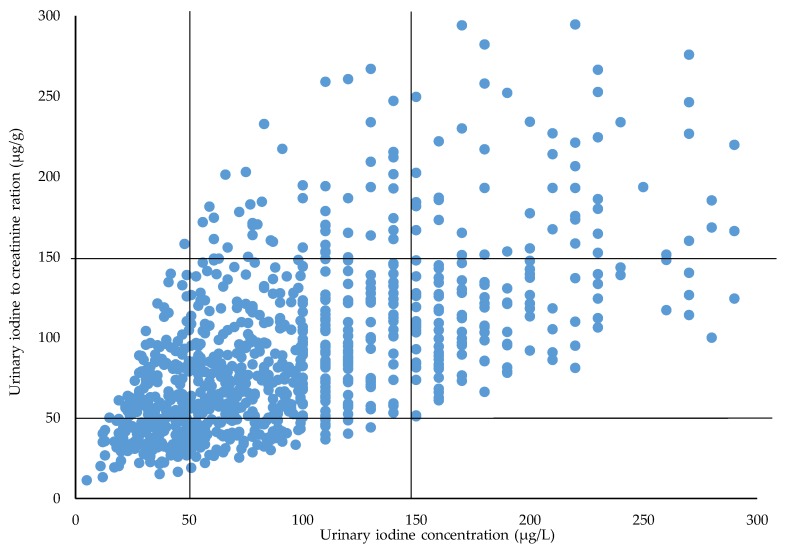
Assignments of the pregnant women into tertiles of urinary iodine concentration (µg/L). (Tertile 1; 0–50 µg/L, tertile 2: >50–150 µg/L, and tertile 3: >150 µg/L). Values above 300 are not included in the figure (*n* = 30).

The correlation between UIC (µg/L) and UI/Cr (µg/g) was high and significant (Spearmans rho 0.65). Figure 3 illustrates that the participants were assigned in the same or in the adjacent tertile, and that only one participant was grossly misclassified (i.e., UIC <50 µg/L and UI/Cr of >150 µg/L). The agreement of tertiles assignment between UIC and UI/Cr was 63.4% in the samples when the UIC was divided into <50 µg/L, ≥50–150 µg/L, and >150 µg/L (Figure 3). Of the 221 participants with UIC <50 µg/L, 107 (48.4%) had UI/Cr ≥50 µg/g (Figure 3). Oppositely, 212 participants had UI/Cr <50 µg/g and 93 participants had UIC ≤50 µg/L. In summary, 111 participants had both UIC and UI/Cr <50 µg/L. The agreement of dichotomization of assignment between UIC and UI/Cr ratio showed that 83.9% were correctly assigned into the same category when UIC was divided into ≤150 µg/L and >150 µg/L (Figure 3).

### 3.4. Association between Iodine Status and Maternal Characteristics

In linear regression analyses using UIC as the dependent variable, three variables were significantly related to UIC; use of iodine supplement, intake of dairy products, and maternal residence. (Table 4). The same linear regression model using UI/Cr as the dependent variable showed a positive significant linear relationship between UI/Cr and use of iodine supplement, intake of dairy products, trimester, and time (hour) of urine sampling (Table 5). Additional adjustments of other factors were not significant and were excluded from the model (*p* > 0.05). The associations with use of iodine supplement and intake of dairy products were related to the level of iodine in both regression models.

## 4. Discussion

The main finding of this study was that median UIC was 85 µg/L, which is below the recommended cut off value of 150 µg/L set by WHO [8]. This suggests that many of the pregnant women in this study had an insufficient iodine intake. In fact, almost 80% of the women had a UIC <150 µg/L. Dietary factors such as use of iodine supplements and intake of dairy products, as well as residence of the mother and season of urine sampling, were all significantly associated with both UIC and UI/Cr. The agreement of correct assignments between UIC and UI/Cr was 84% when the UIC was dichotomized (<150 µg/L and >150 µg/L); 111 women (11.5%) had both UIC and UI/Cr <50 µg/L. To our best knowledge, this is the first survey using UIC and UI/Cr in such a large sample of pregnant women living in several areas in Norway.

Insufficient iodine nutrition in pregnant women has been demonstrated in several European countries. In studies from Austria [11], Belgium [25], Denmark [9], Latvia [26], Poland [12], Portugal [27], Spain [28], Sweden [10], and the UK [19], the median UIC was in the range from 57 µg/L in UK to 140 µg/L in Spain. In contrast, only a few studies have reported sufficient iodine intake in pregnant women. In Iceland, the median UIC was 200 µg/L [29] and in the Netherlands UIC was 230 µg/L in pregnant women [30]. In studies from Norway, median UIC was 92 µg/L (*n* = 777) in pregnant women living in the Oslo area from 2016 [15], 84 µg/L (*n* = 197) in the Northern Mother-and-Child contaminant cohort (MISA) from 2007–2009 [14], and 69 µg/L in the MoBa cohort (*n* = 119) from 2003–2004 [13]. In the MISA study and in the MoBa cohort, 80% and 89%, respectively, of the pregnant women had UIC <150 µg/L. The UIC levels in the Oslo area, MISA, MoBa, and our study are in line with results from the estimated dietary iodine intake in more than 60 thousand mothers in the MoBa cohort study [13]. In the MoBa study, 54.3% had an estimated iodine intake below the Nordic recommendation of 175 µg/day and only 21.7% of the women reached the WHO recommendation of 250 µg iodine/day. In the Oslo area study, 55% had a calculated iodine intake below estimated average requirement (EAR) of 160 µg/day [15]. Our study, as was also the case for the estimated iodine intake in the MoBa study [13] and the study in the Oslo area [15], comprised many participants and therefore suggest that pregnant women in Norway are at risk of having insufficient iodine intake. 

Even though several factors determine the level of iodine in urine, the UIC captures the total iodine intake from all dietary sources [7]. In our study, we found a strong relationship between UIC or UI/Cr and iodine supplement intakes and the intake of milk and dairy products. In studies from Denmark [9], Poland [12], and the UK [19] it was also reported a higher UIC or UI/Cr in pregnant women using iodine supplements and/or consumed ≥2 portions of milk and dairy products per day. The MoBa study has also shown that the estimated dietary iodine intake was strongly associated with the intake of iodine supplement, milk and dairy products, and seafood [13]. Since fortification of cow fodder started around 1950 in Norway, milk and dairy products have, been the most important dietary iodine source for Norwegians [31]. This was confirmed in a study using dietary data from a representative sample of Norwegians [32], and by the MoBa study using dietary data of pregnant women [13]. However, the total iodine intake is still not in accordance with the recommendation. Analysis of the iodine content in milk and dairy products in Norway in 2000 [33] showed a seasonal variation, but more recent analyses show that this variation has been less pronounced in recent years [34,35]. The urine samples in the present study were collected in 2011–12. Although we found a seasonal effect of UIC, meaning that UIC in samples collected in the winter (December–February) was significantly higher than in autumn (September–November), this difference was not related to a higher intake of milk and dairy products or supplement intake among these women. Therefore, it is reasonable to speculate that the iodine level in the milk and dairy products was higher during the winter season at the time of the data collection in the present study (i.e., 2011–12). As to residence, the women living in the eastern part of Norway with significantly higher UIC, did not have a higher milk and dairy or supplement intake than women living in the three other parts of Norway (mid, north, or west). Even if we found these higher UIC related to season and residence, all UIC levels were below the cut off value of ≤150 µg/L. 

Although, seafood intake contributes to the iodine intake, the study among pregnant women in Iceland is the only one showing that fish intake of ≥2 times/week results in UIC above 150 µg/L [29]. The intake of haddock (lean fish) was reported by 83% of Icelandic women. The iodine content within and between fish species varies, and in general, lean fish has higher levels of iodine than fatty fish [32,35]. The lack of associations between UIC or UI/Cr and intake of seafood in the present study is most likely due to the fact that approximately 30% of the women reported to consume seafood in accordance to the recommendation of 300–450 g/week. However, since the urine reflects only the last day of iodine intake, it would be more difficult to find a correlation with seafood intake which is eaten 2–3 times per week than milk and dairy products which are consumed more on a daily basis.

UIC is not recommended to be applied at an individual level. By using UIC and UI/Cr, respectively, for the classification of iodine deficiency, one may inadvertently come to classify women into different groups. By using UIC, our results showed that 221 (23.2%) of the women were classified with an UIC below 50 µg/L. By using the UI/Cr, 212 (22.2%) were so classified. Although the number of women is quite similar by both methods, only 111 individuals were assigned to the same group, i.e., had both UIC and UI/Cr <50 µg/L. This is a caveat to the effect that one should be careful when selecting the classification criterion. The inter- and intra-individual variation in UIC is caused by variation in fluid intake as well as differences in iodine intake. Since the creatinine excretion is relatively constant, the use of creatinine adjustments can minimize the variation in UIC caused by variation in the urine volume [36]. In the study by Henjum et al. 2018 [15] a sub-study group of 49 pregnant women had a median urine volume of 1.4 L. Considering this, we have overestimated the proportion of pregnant women having an UIC <150 µg/L when using the WHO criteria [8].

The relationship of UIC or UI/Cr to maternal characteristics shows conflicting results. We found that trimester, time of the day of urine sampling, smoking, parity, education, and income were significantly associated with the UI/Cr (*p* < 0.01) but not with the UIC. Studies using UIC have reported a reduction of UIC throughout pregnancy [17,20,37,38] or an increase of UIC [19,28]. UIC in our study increased with trimester, however not significantly. Studies using UI/Cr also report increasing level of UI/Cr by increasing gestational week [19,25,26] and others have reported decreasing level of UI/Cr throughout pregnancy [39].

This study has several strengths. Since the women were recruited year-round, the seasonal bias noted in other studies was reduced. The women were recruited from several regions in Norway and this is the first study using spot urine samples in a large sample of pregnant women. UIC is the recommended marker of assessments of iodine nutrition in population studies by WHO, and with almost 1000 samples we were able to estimate the iodine level within a precision range of ±5% [36]. Further, analysis of iodine was performed with ICP-MS, which is a highly accurate method. The high retention rate is a strength to this study. This was possibly due to the participants being followed up by health care nurses serving as research assistants. One limitation is that most of the samples were collected in the second, and not in first trimester. It should be noted that the project is not a study of a truly representative population in terms of socioeconomic background. However, the study population is sufficiently large to conclude that the median UIC or UI/Cr indicate insufficient iodine intake in the population [40].

Insufficient iodine intake among pregnant women is widespread in Europe. The lack of knowledge about the importance of iodine and about dietary sources of iodine among women can be regarded as a risk factor for iodine deficiency [41]. More attention by health care providers, including obstetricians and midwives, may be important in solving this problem, along with the identification of new strategies capable to increase the knowledge of and awareness in the general population [42]. Today, there is no national monitoring of iodine status of the Norwegian population, but according to a national plan of action for a better diet, public authorities should follow-up initiatives to improve and secure the iodine intake [43]. Iodized salt is the most important source worldwide and is the agreed strategy for achieving iodine sufficiency [8]. In Norway, iodized salt is available as table salt, however the level of iodine is only 5 µg/g, and the food industry is not permitted to use iodized salt. To improve the situation in Norway, the health authorities have recently initiated a risk–benefit analysis of increased iodization of table salt and iodization of bread products. Another action is to recommend iodine supplementation to pregnant women and other vulnerable groups with low consumption of milk. 

## 5. Conclusions

These findings may potentially be of great public health importance, suggesting that a large proportion of pregnant women in Norway is iodine deficient. Further studies should investigate to what extent inadequate iodine status during pregnancy influences developmental skills in infancy, as well as the thyroid function of the mothers. In conclusion, this study adds to the increasing evidence that pregnant women in Norway are iodine deficient and that the diet of pregnant women does not secure a sufficient iodine intake. There is an urgent need for public health strategies to secure adequate iodine nutrition among pregnant women in Norway.

## Figures and Tables

**Table 1:** Urinary iodine concentration (µg/L (UIC)) and urinary iodine to creatinine ratio (µg iodine/g creatinine (UI/Cr ratio)) according to maternal demographic and behavioral characteristics given as median with 25th and 75th percentiles in the study population.

	*n*	UIC (µg/L)	*p*-Value *	UI/Cr Ratio (µg/g)	*p*-Value *
Maternal age, years	937		0.429		0.100
<25	114	80 (53–143)		71 (46–124)	
25–29	348	85 (50–130)		79 (49–122)	
30–34	305	83 (54–120)		86 (59–121)	
≥35	170	88 (51–160)		86 (54–139)	
Smoking (daily) while pregnant	954		0.128		0.019
No	882	85 (57–140)		83 (54–125)	
Yes	72	70 (44–120)		64 (45–107)	
Parity	954		0.846		0.034
0	547	78 (44–130)		90 ^a^ (55–138)	
1	337	82 (52–135)		80 ^a,b^ (52–119)	
≥2	120	80 (43–130)		75 ^b^ (45–118)	
Educational level	954		0.217		<0.0001
Primary ^1^/high school	231	78 (48–130)		73 ^a^ (45–121)	
College/University < 4 year	360	85 (47–140)		82 ^a,b^ (53–123)	
College/University ≥ 4 year	413	78 (47–130)		90 ^b^ (59–134)	
Cohabitation	954		0.632		0.844
Living with partner	912	79 (47–130)		83 (53–127)	
Not living with partner	42	85 (47–130)		83 (50–139)	
Ethnicity	954		0.126		0.229
Norwegian	945	78 (47–130)		82 (53–127)	
Other than Norwegian	59	92 (56–140)		90 (59–121)	
Income (mother)	954		0.331		0.007
<300,000 NOK	320	78 (45–130)		82 ^a^ (50–124)	
300,000–449,000 NOK	453	80 (47–130)		80 ^a^ (51–126)	
≥450,000 NOK	231	79 (50–140)		94 ^b^ (60–138)	

^1^ 31 participants have completed primary school. Different letters indicate statistical differences. * *p*-value for differences between groups using Mann-Whitney U to compare two groups or Kruskall–Wallis test comparing categories with more than two groups.

**Table 2:** Urinary iodine concentration (µg/L (UIC)) and urinary iodine to creatinine ratio (µg iodine/g creatinine (UI/Cr ratio)) according to maternal sample collection characteristics given as median with 25th and 75th percentiles in the study population.

	*n*	UIC (µg/L)	*p*-Value *	UI/Cr Ratio (µg/g)	*p*-Value *
Trimester	924		0.052		0.003
First (0–13 week)	10	66 (41–155)		87 ^a^ (44–105)	
Second (14–27 week)	674	83 (50–130)		79 ^a^ (50–119)	
Third (28–44 week)	240	92 (57–150)		94 ^b^ (60–145)	0.001
Residence in Norway	954		<0.0001		<0.0001
North	144	82 ^b^ (49–128)		83 ^a^ (52–113)	
Mid	255	72 ^a^ (44–110)		66 ^a^ (47–107)	
West	217	83 ^b^ (53–130)		75 ^a^ (48–112)	
East	338	100 ^b^ (61–150)		98 ^b^ (64–147)	
Time of urine sampling	949		0.283		<0.0001
Morning (06 a.m.–10 a.m.)	262	79 (50–120)		72 ^a^ (47–105)	
Noon (10 a.m.–02 p.m.)	305	87 (54–140)		81 ^a,c^ (50–117)	
Afternoon (02 p.m.–07 p.m.)	299	90 (54–140)		95 ^b^ (59–142)	
Evening/night (07 p.m.–06 a.m.)	35	90 (40–150)		107 ^b^ (58–148)	
Season of urine sampling	899		0.006		0.006
Winter (Dec.–Feb.)	264	91 ^a^ (51–140)		93 ^a^ (58–132)	
Spring (Mar.–May.)	239	90 ^a^ (55–140)		86 ^a,b^ (53–126)	
Summer (Jun.–Aug.)	132	85 ^a,b^ (50–130)		76 ^b^ (44–120)	
Autumn (Sep.–Nov.)	264	73 ^b^ (46–120)		74 ^b^ (52–114)	

Different letters indicate statistical differences. * *p*-value for differences between groups using Mann–Whitney U to compare two groups or Kruskall–Wallis test comparing categories with more than two groups.

**Table 3:** Urinary iodine concentration (µg/L (UIC)) and urinary iodine to creatinine ratio (µg iodine/g creatinine (UI/Cr ratio)) according to selected maternal dietary intake given as median with 25th and 75th percentiles in the study population.

	*n*	UIC (µg/L)	*p*-Value *	UI/Cr Ratio (µg/g)	*p*-Value *
Dietary factors					
Seafood dinner	934		0.539		0.103
<2 portions/week	653	78 (48–130)		82 (51–126)	
≥2 portions/week	281	80 (47–125)		88 (57–129)	
Egg	821		0.430		0.156
<3 eggs/week	701	80 (49–130)		82 (53–127)	
≥3 eggs/week	120	78 (36–140)		92 (55–145)	
Dairy products	806		<0.0001		<000.1
0–1 portion/day	153	57 ^a^ (39–110)		64 ^a^ (45–110)	
2–3 portion/day	524	83 ^b^ (48–130)		83 ^b^ (55–130)	
4–9 portions/day	128	99 ^b^ (59–168)		105 ^c^ (77–150)	
Iodine supplement use	948		<0.0001		<0.0001
Yes (≥5 times/week)	144	120 ^a^ (72–208)		140 ^a^ (84–233)	
No (<5 times/week)	804	75 ^b^ (44–120)		78 ^b^ (51–116)	

Different letters indicate statistical differences. * *p*-value for differences between groups using Mann–Whitney U to compare two groups or Kruskall–Wallis test comparing categories with more than two groups.

**Table 4:** Predictors of urinary iodine concentration (UIC) in pregnant women with UIC (µg/L) as dependent variable.

Variable	B	SE_B_	β
Intercept	43.963	12.961	
Supplement user	61.839	7.601	0.287 *
Dairy product intake	16.124	4.909	0.116 *
Residence	7.230	2.715	0.94 *

* *p* < 0.05; B = unstandardized regression coefficient; SE_B_ = Standard error of the coefficient; β = standardized coefficient.

**Table 5:** Predictors of urinary iodine to creatinine ratio (UI/Cr) in pregnant women with UI/Cr (µg iodine/g creatinine) as dependent variable.

Variable	B	SE_B_	β
Intercept	−28.988	19.443	
Supplement user	87.751	7.955	0.381 *
Dairy product intake	19.38	5.149	0.131 *
Time of urine sampling	15.633	3.461	0.157 *
Trimester	21.283	6.82	0.108 *

* *p* < 0.05; B = unstandardized regression coefficient; SE_B_ = Standard error of the coefficient; β = standardized coefficient.

## Abbreviations

EAR	Estimated average requirement
LiN	Little in Norway study
MoBa	The Norwegian Mother and Child Cohort Study
MISA	Northern mother-and-child contaminant cohort
UIC	Urinary iodine concentration
UI/Cr	Urinary iodine-to-creatinine ratio
UIE	24-h excretion of iodine
WHO	World Health Organization
